# Methyl Jasmonate Was Involved in Hydrogen Sulfide-Alleviated Cadmium Stress in Cucumber Plants Through ROS Homeostasis and Chlorophyll Metabolism

**DOI:** 10.3390/ijms26020475

**Published:** 2025-01-08

**Authors:** Lijuan Niu, Haixia Zhao, Yunlai Tang, Bo Zhu, Yanshuo Zhao, Qian Wang, Jian Yu

**Affiliations:** School of Life Science and Engineering, Southwest University of Science and Technology, Mianyang 621010, China; niulj0508@163.com (L.N.); 15183996783@163.com (H.Z.);

**Keywords:** cadmium stress, hydrogen sulfide, methyl jasmonate, ROS, photosynthesis

## Abstract

Cadmium (Cd), as one of the most toxic nonessential elements, severely prohibits plant growth and development. Hydrogen sulfide (H_2_S) and methyl jasmonate (MeJA) play essential roles in plant response to abiotic stress. However, the potential mechanism of H_2_S and MeJA in alleviating Cd stress in plants remains unclear. In the current study, the importance and crosstalk of H_2_S and MeJA in the Cd tolerance of cucumber seedlings have been investigated. Our results revealed that Cd stress obviously prohibited the growth of cucumber seedlings. Optimal concentrations of H_2_S donor sodium hydrosulfide (NaHS) or MeJA treatment, respectively, or in combination, significantly enhanced seedling growth under Cd stress. However, the positive effects of H_2_S during seedling growth were obviously reversed by the application of MeJA biosynthesis inhibitors, which implied that MeJA might be involved in the H_2_S-improved growth of cucumber seedlings under Cd stress. Moreover, Cd stress resulted in the increase in hydrogen peroxide (H_2_O_2_), superoxide radical (O_2_^·−^) accumulation, and impaired the functioning of the ascorbate–glutathione cycle. Both H_2_S and MeJA decreased the reactive oxygen species (ROS) level and ameliorated the negative effects of Cd stress through significantly increasing the ratio of ascorbate (AsA)/dehydroascorbic acid (DHA) and reduced glutathione (GSH)/oxidized glutathione (GSSG). Besides that, the expression level of ROS scavenge genes was significantly upregulated by the application of exogenous H_2_S or MeJA treatment. Moreover, H_2_S and MeJA significantly enhanced the chlorophyll concentration and inhibited chlorophyll degradation through decreasing the expression levels of chlorophyll catabolic enzymes. Additionally, exogenous H_2_S and MeJA obviously enhanced the chlorophyll fluorescence. However, MeJA biosynthesis inhibitors significantly suppressed the positive role of H_2_S. The above results suggested MeJA is involved in H_2_S-induced Cd stress alleviation in cucumber seedlings through enhancing ROS-scavenge capacity and improving the photosynthesis system.

## 1. Introduction

Hydrogen sulfide (H_2_S) has been considered as the third gaseous signaling molecule after nitric oxide and carbon monoxide [1]. In plants, L/D-cysteine desulfhydrase (L/DCD) is considered to be as primarily responsible for generating H_2_S [2]. Numerous studies have shown that H_2_S plays an essential role in regulating plant growth and development such as seed germination [3], the rooting process [4], flowering [5], fruit ripening, and senescence [6]. Moreover, emerging evidence has suggested that H_2_S is involved in the various physiological processes of plants, including stomatal closure [7], photosynthesis [8], and so on. Furthermore, research on the role of H_2_S in plants has mainly focused on the protective effect against abiotic stress, such as drought stress [9], cold stress [10], salt stress [11], and heavy metal stress [12]. For example, in *Trigonella foenum-graecum*, exogenous H_2_S enhanced tolerance to cadmium (Cd) stress through increasing the activities of antioxidant enzymes and modulating polyamine content [13]. Moreover, H_2_S treatment significantly decreased Cd accumulation and reactive oxygen species (ROS) production in *S. matsudana*; meanwhile, it restored the redox status of ascorbate (AsA) and reduced glutathione (GSH) through increasing monodehydroascorbate reductase (MDHAR) and dehydroascorbate reductase (DHAR) activities under Cd stress [14]. Furthermore, it has been demonstrated that the exogenous application of H_2_S significantly alleviated Cd stress by increasing the photosynthesis performance [8]. These results implied that H_2_S as positive regulator is involved in plant response to Cd stress.

Jasmonic acid (JA) and the related compound methyl jasmonate (MeJA), which are collectively referred to as Jasmonates (JAs), function in regulating plant growth and development, and also emerge as crucial cellular regulators involved in various physiological and biochemical processes, including stress responses, secondary metabolite synthesis, and so on [15,16]. Emerging evidence has shown that MeJA enhances plant tolerance under a variety of abiotic stresses. For example, the application of MeJA increased salt tolerance in okra through regulating endogenous hormones metabolism, osmotic adjustment, the process of photosynthesis, and ROS metabolism [17]. Moreover, exogenous MeJA also improved drought stress tolerance through regulating water use efficiency [18]. Additionally, it has been reported that exogenously applied MeJA alleviated Cd damage by improving the antioxidative ability. For instance, MeJA significantly reduced Cd damage through enhancing S-assimilation and GSH level and subsequently enhanced photosynthesis in mustard [19]. Meanwhile, MeJA could alleviate the Cd toxicity of wheat through enhancing the peroxidase (POD), DHAR, MDHAR, and glutathione reductase (GR) activities and regulating the expression levels of Cd transporter genes [20]. Also, MeJA played a vital role in enhancing the tolerance to cadmium by regulating photosynthesis [21]. Consequently, MeJA plays an essential role in plant growth and abiotic stress response. However, the mechanism of JA signaling in the plant response to abiotic stress still needs further exploration.

Increasing studies have proven that there existed a crosstalk between MeJA and H_2_S signaling in plants. For example, Deng et al. [22] found that H_2_S could function as a downstream molecule of JA signaling to inhibit stomatal development of *Arabidopsis*. Also, the inhibition of MeJA biosynthesis significantly declined the melatonin-induced endogenous H_2_S production of watermelon [23]. In addition, it has been reported that H_2_S obviously increases the endogenous JA level through inducing the gene expression of the JA pathway and increases the resistance to soft rot in kiwifruit during storage [24]. Moreover, Yu et al. [25] indicated that H_2_S could mediate MeJA signaling to alleviating chilling injury in peach. Additionally, Tian et al. [26] found that pretreatment with MeJA significantly reduced Cd damage, and H_2_S has been proven to participate the MeJA-induced Cd tolerance in *foxtail millet*. These studies suggested a possible crosstalk between MeJA and H_2_S signaling in plants. However, the relationship between MeJA and H_2_S in the enhancement of Cd tolerance in cucumber seedlings is still not clear. In this study, we hypothesis that (1) exogenous H_2_S or MeJA treatment could mitigate Cd-induced oxidative damage in cucumber seedlings through improving ROS-scavenge capacity and enhancing the photosynthesis system. (2) MeJA is involved in H_2_S-enhanced Cd stress resistance through regulating ROS homeostasis and chlorophyll metabolism in cucumber seedlings under Cd stress. In order to demonstrate these hypotheses, we provide evidence that MeJA is involved in H_2_S-enhanced Cd stress resistance in cucumber seedlings in order to improve our understanding of the mechanism of H_2_S signaling under heavy metal stress.

## 2. Results

### 2.1. Cd Stress Inhibited the Growth of Cucumber Seedlings

**Figure 1:**
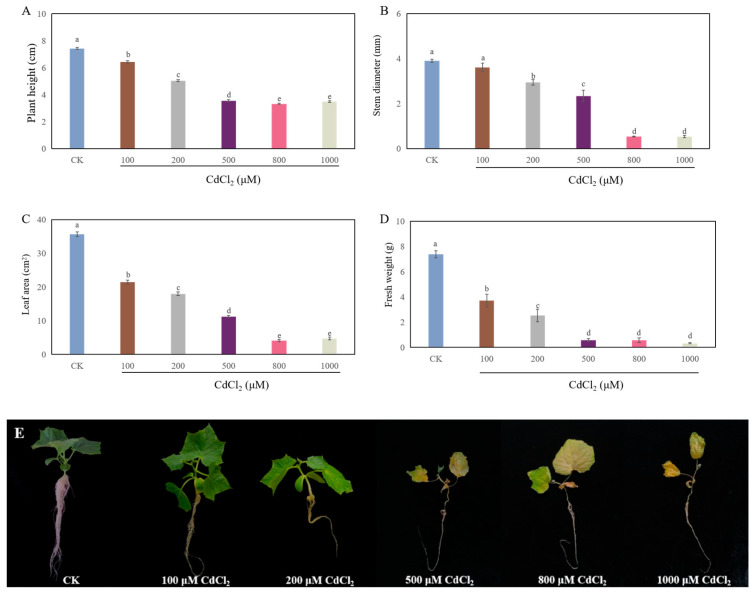
The effect of cadmium chloride (CdCl_2_) at different concentrations on the plant height (**A**), stem diameter (**B**), leaf area (**C**), and fresh weight (**D**) of cucumber seedlings. Photographs (**E**) were taken after 7 days of the treatment indicated. Bars with different letters are significantly different at *p* < 0.05 according to Duncan’s multiple range test.

As shown in Figure 1, different concentrations of cadmium chloride (CdCl_2_) treatment obviously prohibited the growth of cucumber seedlings. Compared to CK, 100 μM CdCl_2_ significantly decreased the plant height, leaf area, and fresh weight by 13.5%, 40.0%, and 49.9%, respectively. Moreover, when these seedlings were treated with 200 μM CdCl_2_, the plant height, stem diameter, leaf area, and fresh weight noticeably declined by 32.2%, 24.4%, 49.5%, and 65.9%, respectively, compared to those of CK. Furthermore, higher concentrations of CdCl_2_ (500 μM, 800 μM, and 1000 μM) caused a more obvious inhibitory effect on the growth of cucumber seedlings. Therefore, 200 μM CdCl_2_ was utilized in the following experiments.

### 2.2. Appropriate Concentrations of H_2_S and MeJA Improved Seedling Growth Under Cd Stress

**Figure 2:**
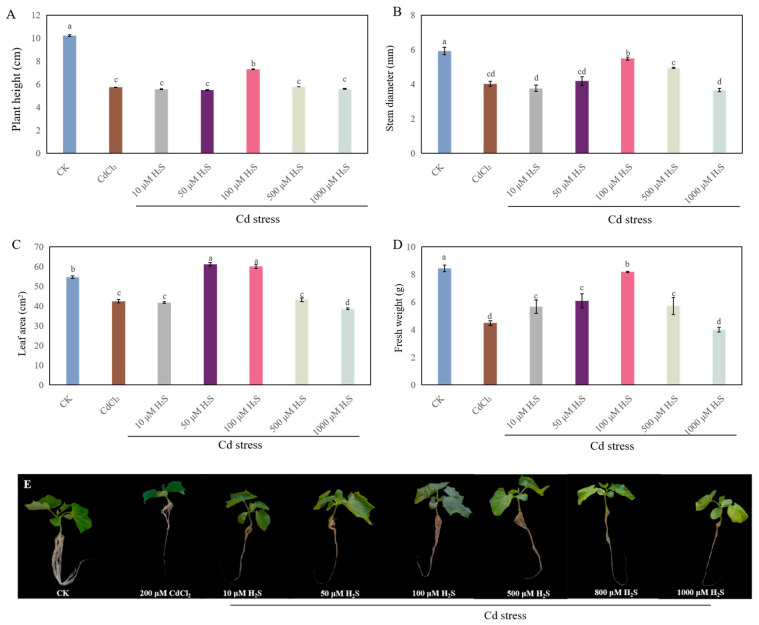
The effects of hydrogen sulfide (H_2_S) at different concentrations on the plant height (**A**), stem diameter (**B**), leaf area (**C**), and fresh weight (**D**) of cucumber seedlings under Cd stress. Photographs (**E**) were taken after 7 days of the treatment indicated. Bars with different letters are significantly different at *p* < 0.05 according to Duncan’s multiple range test.

In order to evaluate the effect of H_2_S on the growth of cucumber seedlings under Cd stress, a dose–response experiment with sodium hydrosulfide (NaHS, H_2_S donor) was performed. As shown in Figure 2, different concentrations of H_2_S treatments under Cd stress significantly affected the growth of cucumber seedlings. There was no marked difference in plant height and stem diameter among CdCl_2_, 10 μM H_2_S, and 50 μM H_2_S. When 100 μM H_2_S was applied, the plant height, stem diameter, leaf area, and fresh weight were significantly increased by 27.6%, 36.4%, 41.4%, and 82.5%, respectively, which is comparable to those of Cd stress treatment (Figure 2). However, higher concentrations of H_2_S (500 μM and 1000 μM) obviously inhibited the seedling growth, implying that the effects of H_2_S on the growth process of cucumber seedlings under Cd stress were dose-dependent. Thus, since 100 μM H_2_S achieved the maximum biological effect during the growth of cucumber seedlings under Cd stress, we utilized 100 μM H_2_S for subsequent experiments.

**Figure 3:**
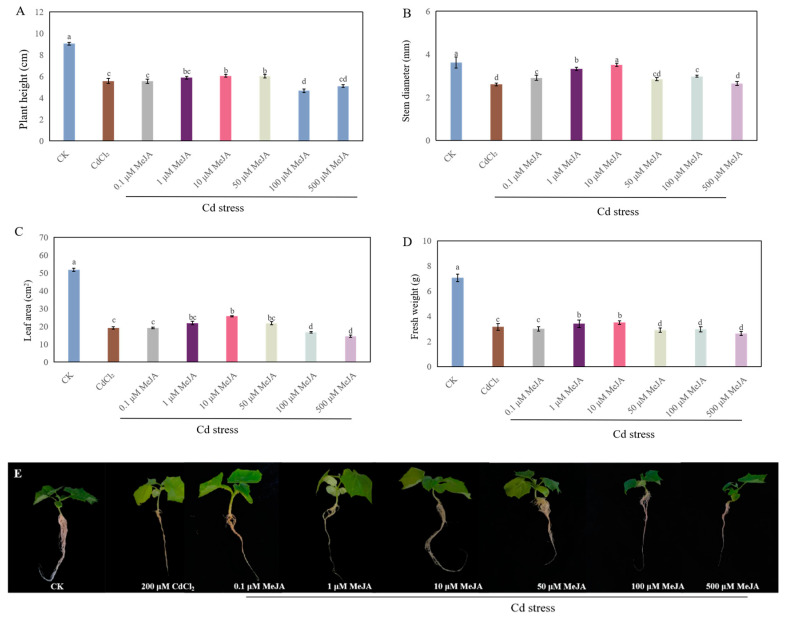
The effect of methyl jasmonate (MeJA) at different concentrations on the plant height (**A**), stem diameter (**B**), leaf area (**C**), and fresh weight (**D**) of cucumber seedlings under Cd stress. Photographs (**E**) were taken after 7 days of the treatment indicated. Bars with different letters are significantly different at *p* < 0.05 according to Duncan’s multiple range test.

As shown in Figure 3, the growth of cucumber seedlings was significantly influenced by different concentrations of MeJA treatment. Compared to CdCl_2_ treatment, 0.1 μM MeJA and 1 μM MeJA obviously increased the stem diameter by 11.9% and 27.7%, respectively (Figure 3B). Moreover, the plant height, stem diameter, leaf area, and fresh weight from 10 μM MeJA treatment increased by 8.4%, 35%, 34.5%, and 11.1%, respectively, compared with those of the CdCl_2_ treatment. Meanwhile, there were no significant differences in plant height and leaf area between 10 μM MeJA and 50 μM MeJA treatment under Cd stress (Figure 3A,C). However, higher concentrations of MeJA (100 μM and 500 μM) significantly hindered the growth of cucumber. These results indicated that 10 μM MeJA remarkably reversed the adverse effects of Cd stress and improved the growth of cucumber seedlings.

### 2.3. Effects of MeJA Biosynthesis Inhibitors on the Growth of Cucumber Under Cd Stress

**Figure 4:**
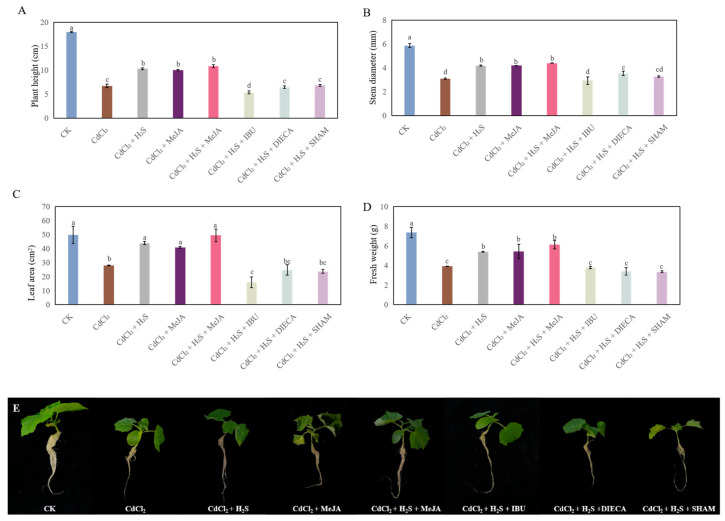
The effects of methyl jasmonate (MeJA) biosynthesis inhibitors on the plant height (**A**), stem diameter (**B**), leaf area (**C**), and fresh weight (**D**) of cucumber seedlings under Cd stress. Photographs (**E**) were taken after 7 days of the treatment indicated. Bars with different letters are significantly different at *p* < 0.05 according to Duncan’s multiple range test.

In order to further investigate the relationship between H_2_S and MeJA in improving the growth of cucumber seedlings under Cd stress, MeJA biosynthesis inhibitors were used in this experiment. As shown in Figure 4, H_2_S, MeJA, or H_2_S + MeJA treatment significantly enhanced the seedlings’ growth under Cd stress. However, the plant height, stem diameter, leaf area, and fresh weight, which were treated with ibuprofen (IBU), significantly declined by 70.1%, 64.0%, and 61.9%, respectively, when compared to CdCl_2_ + H_2_S treatment (Figure 4). Meanwhile, the MeJA biosynthesis inhibitors diethyldithiocarbamic acid (DIECA) and Salicylhydroxamic acid (SHAM) remarkably inhibited H_2_S-improved seedlings’ growth under Cd stress, compared to that of the H_2_S treatment.

### 2.4. Endogenous Hydrogen Peroxide (H_2_O_2_) and Superoxide Radical (O_2_^·−^) Level Under Different Treatments

**Figure 5:**
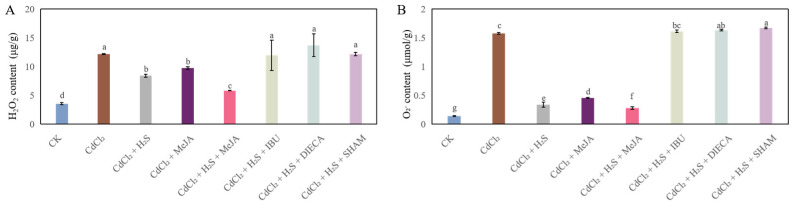
The effects of H_2_S and MeJA on the endogenous hydrogen peroxide (H_2_O_2_, (**A**)) and superoxide radical (O_2_^·−^, (**B**)) level of cucumber seedlings under Cd stress. Bars with different letters are significantly different at *p* < 0.05 according to Duncan’s multiple range test.

Figure 5 shows that the level of H_2_O_2_ and O_2_^·−^ of cucumber seedlings in the Cd treatment significantly increased compared to those of CK. However, the exogenous application of H_2_S or MeJA obviously resulted in a reduction in endogenous H_2_O_2_ and O_2_^·−^ level compared with Cd stress alone. Moreover, seedlings treated with H_2_S + MeJA caused a significant decline in the level of endogenous H_2_O_2_ and O_2_^·−^ compared with those of CdCl_2_ treatment. However, the endogenous H_2_O_2_ and O_2_^·−^ levels of treatment with MeJA synthesis inhibitors were significantly higher than those of the H_2_S or MeJA treatment.

### 2.5. The Ratio of AsA/DHA and GSH/GSSG in Cucumber Seedlings Under Different Treatments

**Figure 6:**
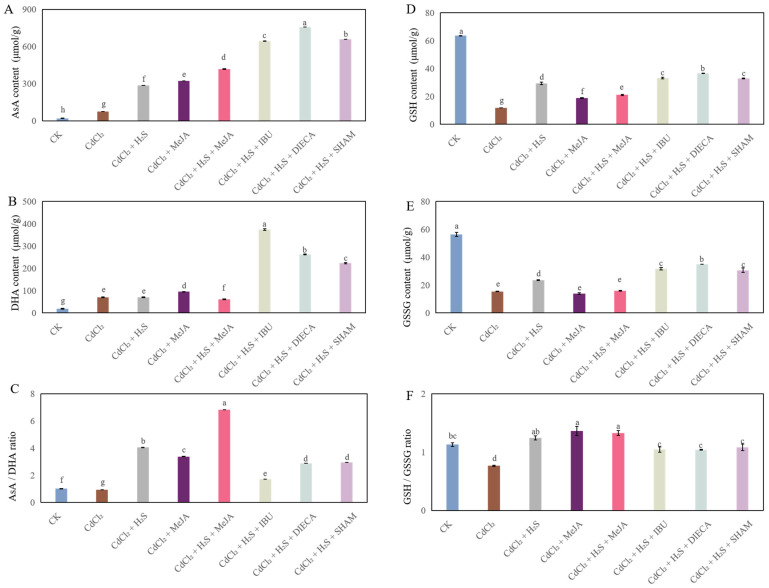
The effects of H_2_S and MeJA on the content of ascorbate (AsA, (**A**)), dehydroascorbic acid (DHA, (**B**)), the ratio of AsA/DHA (**C**), reduced glutathione (GSH, (**D**)), oxidized glutathione (GSSG, (**E**)), and the ratio of GSH/GSSG (**F**) in cucumber seedlings under Cd stress. Bars with different letters are significantly different at *p* < 0.05 according to Duncan’s multiple range test.

As shown in Figure 6A, compared to the control, Cd stress obviously enhanced the AsA content. However, H_2_S, MeJA, or H_2_S + MeJA treatment significantly elevated the level of AsA when compared to that of Cd stress alone. Moreover, the AsA level of these inhibitor treatments is higher than that of H_2_S, MeJA, or H_2_S + MeJA treatment. Meanwhile, DHA content when treated with H_2_S, MeJA or H_2_S + MeJA is less than that of the IBU, DIECA, or SHAM treatment (Figure 6B). CdCl_2_ treatment significantly decreased the AsA/DHA ratio, compared with CK. However, compared to Cd stress, the ratio of AsA/DHA remarkably increased when treated with H_2_S, MeJA, or H_2_S + MeJA. Besides that, a considerable reduction in the AsA/DHA ratio was observed under H_2_S + IBU, H_2_S + DIECA, or H_2_S + SHAM treatment (Figure 6C). Furthermore, H_2_S, MeJA, or H_2_S + MeJA treatment significantly enhanced the GSH content compared to that of the Cd treatment (Figure 6D). Meanwhile, compared to Cd stress, the application of exogenous H_2_S increased the GSSG content in cucumber seedlings, but there was no significant difference in the GSSG level among the treatments of CdCl_2_, CdCl_2_ + MeJA, and H_2_S + MeJA. The effects of MeJA synthesis inhibitors on the GSSG level followed the same pattern as the effect on the DHA level (Figure 6E). Additionally, the GSH/GSSG ratio of treatment with CdCl_2_ is less than that of the control (Figure 6F). However, H_2_S, MeJA, or H_2_S + MeJA treatment significantly enhanced the ratio of GSH/GSSG; meanwhile, seedlings treated with MeJA synthesis inhibitors exhibited a remarkable decline in the ratio of GSH/GSSG in comparison with H_2_S, MeJA, or H_2_S + MeJA treatment (Figure 6F).

### 2.6. The Expression Level of the ROS Scavenge Genes in Cucumber Seedlings Under Different Treatments

**Figure 7:**
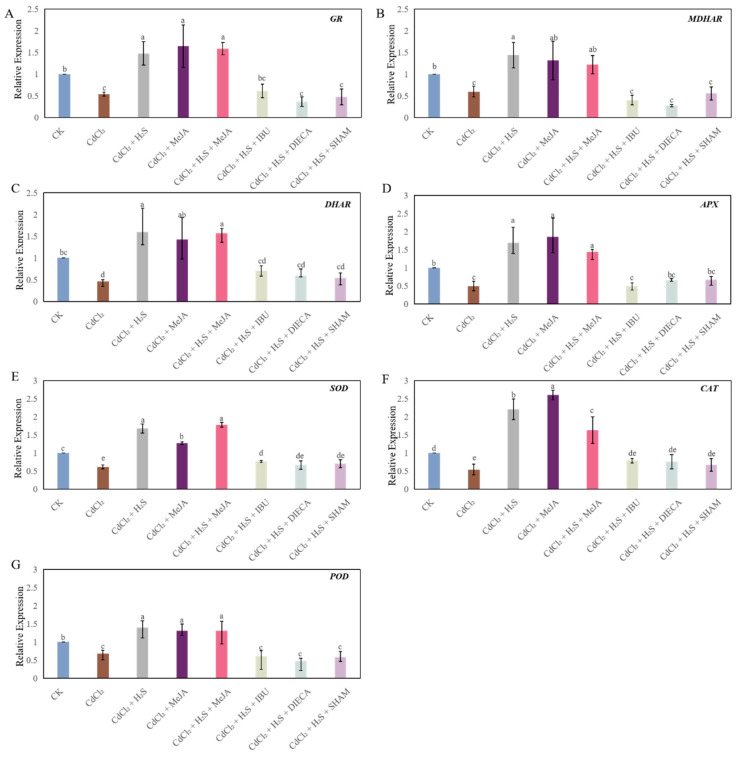
The effects of H_2_S and MeJA on the expression level of glutathione reductase (GR, (**A**)), monodehydroascorbate reductase (MDHAR, (**B**)), dehydroascorbate reductase (DHAR, (**C**)), ascorbate peroxidase (APX, (**D**)), superoxide dismutase (SOD, (**E**)), catalase (CAT, (**F**)), peroxidase (POD, (**G**)) in cucumber seedlings under Cd stress. Bars with different letters are significantly different at *p* < 0.05 according to Duncan’s multiple range test.

As shown in Figure 7, compared to CK treatment, Cd treatment significantly reduced the expression level of GR, MDHAR, DHAR, ascorbate peroxidase (APX), superoxide dismutase (SOD), catalase (CAT), and POD. However, H_2_S, MeJA, or H_2_S + MeJA treatment under Cd stress significantly improved the expression level of these genes, compared to those of the Cd treatment. Nevertheless, treatment with MeJA synthesis inhibitors remarkably down-regulated the transcriptional levels of the ROS scavenge genes compared to those of H_2_S, MeJA, or H_2_S + MeJA treatment (Figure 7).

### 2.7. Chlorophyll Metabolism in Cucumber Seedlings Under Different Treatments

**Figure 8:**
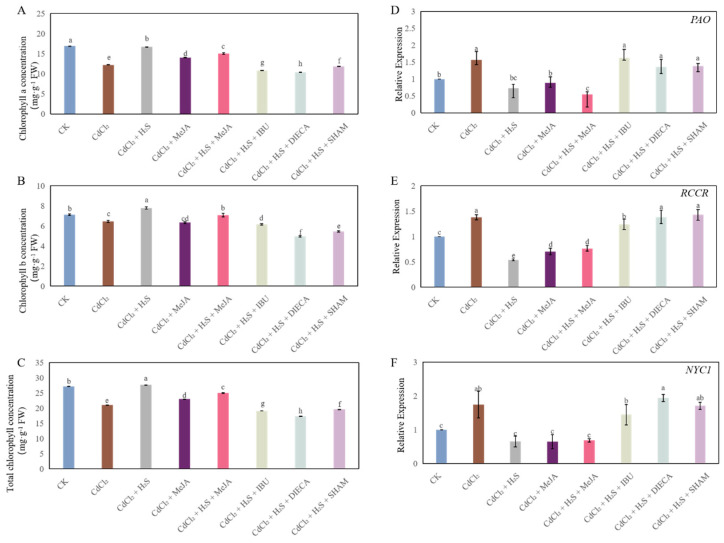
The effects of H_2_S and MeJA on the chlorophyll a concentration (**A**), chlorophyll b concentration (**B**), total chlorophyll concentration (**C**) and the expression levels of pheophorbide a oxygenase (PAO, (**D**)), red chlorophyll catabolite reductase (RCCR, (**E**)), non-yellow coloring 1(NYC1, (**F**)) in cucumber seedlings under Cd stress. Bars with different letters are significantly different at *p* < 0.05 according to Duncan’s multiple range test.

Compared to CK, Cd stress significantly decreased the chlorophyll a (Chl a), chlorophyll b (Chl b), and total chlorophyll (Chl) concentration in leaves of cucumber seedlings. H_2_S, MeJA, or H_2_S + MeJA treatment remarkably increased chlorophyll concentration, compared with those of the CdCl_2_ treatment alone. However, IBU, DIECA, or SHAM treatment obviously decreased the Chl a, Chl b, and total Chl concentration, compared to H_2_S, MeJA, or H_2_S + MeJA treatment (Figure 8A–C). Moreover, the relative expression levels of Chl catabolic genes were determined. As shown in Figure 8D–F, Cd stress dramatically up-regulated the expression level of pheophorbide a oxygenase (*PAO*), red chlorophyll catabolite reductase (*RCCR*), non-yellow coloring 1 (*NYC1*). However, the application of exogenous H_2_S and MeJA markedly decreased the *PAO*, *RCCR*, *NYC1* expression level compared with that of Cd treatment. Interestingly, IBU, DIECA, or SHAM treatment obviously reversed the effect of H_2_S on the transcriptional levels of Chl catabolic enzymes (Figure 8D–F).

### 2.8. Change in Chlorophyll Fluorescence in Cucumber Seedlings Under Different Treatments

As shown in Table 1, compared with CK, Cd stress significantly decreased the values of the maximum quantum yield of PSII (Fv/Fm), the effective quantum yield of PSII (ΦPSII) and photochemical quenching (qP) to 5.56%, 25.68%, and 34.62%, respectively. However, the apparent increase in Fv/Fm, ΦPSII, and qP was observed under H_2_S, MeJA, or H_2_S + MeJA treatment, compared to those of the Cd stress conditions. By contrast, the application of MeJA synthesis inhibitors obviously reduced the Fv/Fm, ΦPSII, and qP values. Moreover, CdCl_2_ treatment remarkably increased the value of non-photochemical quenching (NPQ) compared with that of CK. However, H_2_S, MeJA, or H_2_S + MeJA treatment obviously decreased NPQ. On the contrary, seedlings treated with MeJA synthesis inhibitors showed increased NPQ (Table 1).

## 3. Discussion

Several studies have shown that Cd stress has a significant inhibitory effect on the growth of plant seedlings [27,28]. In our study, Cd stress significantly hindered the growth of cucumber seedlings (Figure 1). A previous study has suggested that exogenous H_2_S alleviates the Cd stress-induced damage by increasing the activity of antioxidant enzymes and the endogenous polyamine level, as well as decreasing the H_2_O_2_ generation and electrolyte leakage of *Trigonella foenum-graecum* [13]. Moreover, it has been reported that MeJA alleviates the Cd toxicity of wheat seedlings by enhancing the antioxidant defense system and decreasing Cd transport [20]. Our results indicated that exogenous H_2_S or MeJA had a dose-dependent effect on promoting the growth of cucumber seedlings under Cd stress (Figure 2 and Figure 3). There are several pieces of evidence which imply that there exists a crosstalk between H_2_S and MeJA during the plant response to abiotic stress [23,26]. For instance, Yu et al. [25] found that H_2_S served as a downstream signaling pathway for MeJA to alleviate chilling injury in peach fruit. Besides that, Su et al. [23] indicated that H_2_S signaling relied on MeJA during the melatonin-induced defense response of watermelon. The inhibition of MeJA biosynthesis obviously decreased melatonin-stimulated H_2_S accumulation. Therefore, in order to further investigate the relationship between H_2_S and MeJA under Cd stress, cucumber seedlings were treated with MeJA biosynthesis inhibitors. Our results indicated that the application of MeJA biosynthesis inhibitors significantly inhibited the H_2_S-promoted growth of cucumber seedlings under Cd stress (Figure 4). These results implied that MeJA might be as a downstream signaling molecule of H_2_S, enhancing the cucumber seedling growth under Cd stress.

Excessive ROS caused oxidative damage to plants under stress conditions. Previous results revealed that Cd treatment significantly elevated the O_2_^·−^ production rate and endogenous H_2_O_2_ content of mulberry leaves [29]. Meanwhile, Ou et al. [27] found that Cd stress increased the ROS production and lipid peroxidation of *Platycladus orientalis* seedlings. Our results indicated that Cd stress significantly elevated the endogenous H_2_O_2_ and O_2_^·−^ levels, further aggravating the oxidative damage. Conversely, H_2_S and MeJA significantly reduced the H_2_O_2_ and O_2_^·−^ accumulation (Figure 5). A previous study found that H_2_S could alleviate oxidative stress through scavenging ROS production [30,31]. Moreover, MeJA treatment decreased H_2_O_2_ and O_2_^·−^ production under abiotic stress [32]. Our results suggested that H_2_S or MeJA maintain lower ROS levels and alleviate the cell damage caused by stress conditions [31]. However, the application of MeJA inhibitors maintained a higher level of H_2_O_2_ and O_2_^·−^ than those of the H_2_S treatment (Figure 5), implying that MeJA is involved in H_2_S-enhanced Cd tolerance of cucumber seedlings.

The ascorbate–glutathione (AsA-GSH) cycle plays an essential role in the antioxidant defense system in plants [33]. The ratios of GSH/GSSG and AsA/DHA indicate the intracellular redox potential, and they are of great importance in the regulation of ROS [34]. Previous research suggested that the toxic impact of Cd stress is accompanied by a reduction in the AsA/DHA ratio in rice leaves [35]. Moreover, the GSH/GSSG ratio of *Arabidopsis thaliana* under Cd stress conditions was lower than that of the control treatment [36]. Our results revealed that the AsA/DHA and GSH/GSSH ratios significantly decreased under Cd stress (Figure 6), which led to a disturbance of the ROS homeostasis. It has been reported that H_2_S exerted its antioxidant activity under stress conditions through enhancing the AsA and GSH levels [37]. Moreover, Kaya et al. [38] found that the application of NaHS improved the proportion of AsA/DHA in the wheat plants under salt stress. In addition, a significant rise in the levels of AsA and GSH involved in the AsA-GSH cycle was detected under MeJA treatment under salt stress [17]. In our study, exogenous H_2_S and MeJA significantly increased the contents of AsA and DHA, along with the AsA/DHA and GSH/GSSH ratios (Figure 6), implying H_2_S and MeJA have a positive effect on mitigating oxidative damage caused by Cd stress through regulating the AsA-GSH cycle. However, the positive impact of H_2_S was critically reversed by the application of MeJA inhibitors, which indicated that MeJA may be a downstream signaling molecule of H_2_S in the regulation of the AsA-GSH cycle of these seedlings under Cd stress. The core enzymes, including ascorbate peroxidase (APX), GR, MDHAR, and DHAR affect the activity of the AsA-GSH cycle through balancing the redox homeostasis under stressful conditions [37]. A previous study found that Cd stress significantly decreased the activities of APX, GR, DHAR, and MDHAR enzymes’ activities [39,40]. Our results showed that Cd treatment significantly down-regulated the transcription levels of *GR*, *MDHAR*, *DHAR*, *APX* in cucumber seedlings (Figure 7A–D), indicating that Cd stress affected the redox balance and caused oxidative damage during the growth of cucumber plants. A previous study suggested that exogenous H_2_S enhances the transcript levels of the *GR*, *MDHAR*, *DHAR*, and *APX* of wheat seedlings under water stress [37]. Moreover, Kaya et al. [38] found that the exogenous application of MeJA increased the activities of AsA-GSH cycle–related enzymes. In our study, exogenous H_2_S and MeJA, applied singly and jointly, significantly up-regulated the expression levels of genes belongs to the AsA-GSH cycle (Figure 7A–D). However, MeJA inhibitors significantly reversed the positive effect of H_2_S, which indicated that MeJA played an essential role in the H_2_S-regulated AsA-GSH cycle under Cd stress. Besides that, significant increases were observed in the expression level of *SOD*, *CAT*, and *POD*, in the leaves of H_2_S or MeJA treatment, compared to those of CdCl_2_ treatment; however, MeJA inhibitors obviously prohibited the effect of H_2_S (Figure 7E–G). These results indicated that MeJA might be involved in an H_2_S-induced increase in ROS-scavenge capacity under Cd stress.

Cd stress led to the inhibition of photosynthesis, causing the decrease in photosynthetic pigment contents and the photosynthetic capacity of chloroplasts [41]. Previous studies have demonstrated that exogenous H_2_S alleviates Cd stress through enhancing the photosynthesis performance [8,42]. Moreover, the application of MeJA increases the tolerance to cadmium by regulating the photosynthesis system [21]. In our study, Cd stress significantly decreased the chlorophyll concentration. However, exogenous H_2_S and MeJA significantly inhibited the decrease in chlorophyll concentration (Figure 8A–C). These results implied that H_2_S and MeJA could effectively prevent the degradation of chlorophyll in cucumber seedlings under Cd stress. Previous results have demonstrated that Chl a is degraded and then converted to the chlorophyll breakdown products by PAO and RCCR [43,44]. Moreover, Yuan et al. [44] supposed that the low expression level of *NYC1* might inhibit the degradation of Chl a. In our study, the application of H_2_S and MeJA obviously down-regulated the expression level of *PAO*, *RCCR*, and *NYC1* (Figure 8D–F). Zhu et al. [45] found that exogenous NaHS treatment significantly decreased the *PAO* expression level for decreasing chlorophyll degradation in celery. Lv et al. [46] found that MeJA treatment significantly decreased the expression levels of *MdPAO6*, *MdPAO8*, and *MdRCCR2*, and also reduced the transcript levels of *MdNYC1* for regulating the Chl degradation of apple. Our present results suggested that H_2_S and MeJA are involved in the regulation of chlorophyll metabolism in cucumber seedlings and significantly prevent the degradation of chlorophyll in cucumber in order to promote photosynthesis under Cd stress. However, MeJA inhibitors had the opposite effects, which implied that endogenous MeJA participated in the H_2_S-regulated chlorophyll metabolism in cucumber seedlings under Cd stress. Chlorophyll fluorescence is a suitable indicator for estimating the change in the photosynthetic performance affected by different stress conditions [47]. In our study, Cd stress treatment significantly decreased the chlorophyll fluorescence. Nevertheless, H_2_S and MeJA treatment significantly enhanced the value of Fv/Fm and ΦPSII under Cd stress, implying that exogenous H_2_S and MeJA could promote electron transport and improve the photochemical efficiency of cucumber seedlings [48]. Moreover, the exogenous application of H_2_S and MeJA improved qP but decreased the value of NPQ (Table 1), suggesting that H_2_S or MeJA could increase the photosynthetic activity of cucumber seedlings under Cd stress through regulating the capacity of the heat-dissipation pathway and alleviating the Cd-induced dissipation of damaging excessive energy [49]. Chen et al. [50] indicated that exogenous H_2_S regulated salt tolerance in *Cyclocarya paliurus* by maintaining chlorophyll fluorescence. In addition, exogenous MeJA obviously enhanced the photosynthetic capacity in plants [41,51]. Our results revealed that the inhibition of endogenous MeJA might reverse the positive effect of H_2_S, enhancing the photosynthetic capacity in cucumber seedlings under Cd stress. These findings suggested that MeJA, as a downstream signaling molecule, is involved in the H_2_S-mediated inhibition of chlorophyll metabolism and the enhancement of photochemical efficiency for improving the tolerance ability of cucumber seedlings under Cd stress.

## 4. Materials and Methods

### 4.1. Plant Material and Growth Condition

Cucumbers (Cucumis sativus cv. ‘Xinchun 4’) were used in our experiment. The seeds were surface-sterilized in 5% sodium hypochlorite for 10 min, washed extensively with distilled water, and then germinated on wet filter paper in Petri dishes at 25 °C for 5 days. Subsequently, cucumber seedlings were transferred into 1/2 Hoagland nutrient solution and plants were grown for a period in a climate chamber with a temperature of 25 °C and a relative humidity of 50–60%, with a 14 h photoperiod of 200 µmols^−1^m^−2^ light intensity. The nutrient solution was changed every 2 days. When the third leaf had just emerged, the seedlings with similar growth status were selected in our experiment. Each treatment was repeated three times.

### 4.2. Experiment Design

The cucumber seedlings of uniform growth were collected for the following treatments with different concentrations for 1 week: CdCl_2_ (Thermo Fisher Scientific, Waltham, MA, USA, 0, 100, 200, 500, 800, 1000 μM), NaHS (Yuanye Bio-Technology Co., Ltd., Shanghai, China, H_2_S donor, 0, 10, 50, 100, 500, 1000 μM), and MeJA (Solarbio, Beijing, China, 0, 0.1, 1, 10, 50, 100, 500 μM). Moreover, MeJA biosynthesis inhibitors ibuprofen (IBU, Yuanye Bio-Technology Co., Ltd., Shanghai, China, 1mM), diethyldithiocarbamic acid (DIECA, Yuanye Bio-Technology Co., Ltd., Shanghai, China, 5 mM), and Salicylhydroxamic acid (SHAM, Sigma-Aldrich, Saint Louis, MO, USA, 2 mM) were used in our study. The concentrations of MeJA biosynthesis inhibitors were based on the results of a preliminary experiment. After the treatments, the plant height and stem diameter of seedlings were measured by vernier calipers. The leaves were scanned with a scanner and the leaf area was analyzed using the Image J 1.8.0 software. The fresh weight was measured using an electronic scale.

### 4.3. ROS Measurement

For H_2_O_2_ measurement, 0.3 g samples were ground in an ice bath with 5 mL of trichloroacetic acid, followed by centrifugation at 12,000× *g* for 15min. A total of 1 mL of clear supernatant was added to 1 mL of potassium phosphate buffer and 2 mL of potassium iodide (Hushi, Shanghai, China). The absorbance of the supernatant was measured at 390 nm [52].

For measuring O_2_^·−^, the samples were ground with potassium phosphate buffer (pH 7.8), and then centrifuged at 4 °C at 8000× *g* for 20 min. The supernatant was added to aminobenzene sulfonic acid (Hushi, Shanghai, China) and α-naphthylamine (Yuanye Bio-Technology Co., Ltd., Shanghai, China), and then reacted at 25 °C for 20 min. The absorbance was measured at 530 nm [53].

### 4.4. Measurement of the Content of AsA, DHA, GSH, GSSG

The content of AsA and DHA were determined as described previously [54] with some modifications. Fresh leaf samples (0.5 g) were homogenized in ice bath with 5% (*V*/*V*) metaphosphoric acid (Hushi, Shanghai, China) and centrifuged at 4 °C at 12,000× *g* for 20 min. Then, the supernatant was used to measure the contents of total ascorbate and AsA. Total ascorbate was measured after incubation in dithiothreitol (Yuanye Bio-Technology Co., Ltd., Shanghai, China) for 10 min. DHA was estimated from the difference in total ascorbate and AsA. GSSG was determined after the removal of GSH by 2-vinylpyridine derivatization. The content of GSH was then estimated by subtracting GSSG from total GSH. The ratios of AsA/DHA and GSH/GSSG were calculated.

### 4.5. Chlorophyll Content and Chlorophyll Fluorescence Measurement

The 0.3 g samples were ground and extracted with 80% acetone (*v*/*v*, Hushi, Shanghai, China). The concentration of chlorophyll a, chlorophyll b, and total chlorophyll content were determined using a spectrophotometer (Shimadzu UV 2550, Kyoto, Japan). The absorbance of samples was recorded at 645 nm and 663 nm [55]. The chlorophyll fluorescence parameters of cucumber leaves were determined using a chlorophyll fluorescence imaging system (Imaging-PAM, Walz, Effeltrich, Germany) at 25 °C. The initial fluorescence yield (Fo), the maximum fluorescence yield (Fm), the effective quantum yield of PSII (ΦPSII), as well as the photochemical quenching (qP) and non-photochemical quenching coefficients (NPQ), were measured after dark adaptation following Genty et al. [56].

### 4.6. Quantative Real-Time PCR Analysis

In order to investigate the effect of H_2_S and MeJA on the ROS-scavenge system and the chlorophyll metabolism pathway in cucumber seedlings under Cd stress, the relative expression of genes encoding for ROS-scavenge enzymes and chlorophyll metabolism enzymes were determined. The expression level of the cucumber *actin* gene was used as an internal control [57]. Total RNA was extracted using the DP419 kit (TianGen, Beijing, China) in accordance with the manufacturer’s instructions. Quantitative real-time PCR experiments were performed using SYBR Green SuperReal PreMix Plus (TianGen, Beijing, China). PCR was initiated at 95 °C for 15 min, followed by 40 cycles of 95 °C for 10 s, and 60 °C for 32 s. The sequences of amplification primers are shown in Table 2. The gene expression was calculated by the 2^−ΔΔCT^ method.

### 4.7. Statistical Analysis

The data were analyzed by using SPSS V. 23.0. The experiments were performed with three independent replicates. Analysis of variance (ANOVA) was carried out, and a value of *p* < 0.05 was considered significantly different according to Duncan’s multiple range test.

## 5. Conclusions

In conclusion, the present study shows that exogenously applied H_2_S and MeJA promoted the growth of cucumber seedlings under Cd stress through reducing the ROS level and improving the antioxidant content and the relative expression levels of the ROS-scavenge gene, thus maintaining the redox status and alleviating the oxidative damage of cucumber seedlings. Moreover, our results also revealed that MeJA might be used as a downstream molecule of the H_2_S signaling pathway to protect the photosynthesis system under Cd stress. These results implied that MeJA was involved in H_2_S-induced Cd stress tolerance in cucumber seedlings. Further research to deepen our understanding of the relationship between H_2_S and MeJA in alleviating plant stress is needed.

## Figures and Tables

**Table 1:** Chlorophyll fluorescence changes in leaves of cucumber seedlings under Cd stress.

Treatments	The Maximum Quantum Yield of PSII (Fv/Fm)	Effective Quantum Yield of PSII (φPSII)	Photochemical Quenching (qP)	Non-Photochemical Quenching (NPQ)
CK	0.72 a	0.74 a	0.78 a	0.24 c
CdCl_2_	0.68 c	0.55 c	0.51 c	0.52 b
CdCl_2_ + H_2_S	0.71 a	0.65 b	0.68 b	0.26 c
CdCl_2_ + MeJA	0.71 b	0.63 b	0.66 b	0.26 c
CdCl_2_ + H_2_S + MeJA	0.72 a	0.64 b	0.70 b	0.36 c
CdCl_2_ + H_2_S + IBU	0.69 c	0.56 c	0.49 c	0.53 b
CdCl_2_ + H_2_S + DIECA	0.68 c	0.44 d	0.47 c	0.74 a
CdCl_2_ + H_2_S + SHAM	0.69 c	0.56 c	0.51 c	0.50 b

Different letters in the same column indicate significant differences among the treatment (*p* < 0.05).

**Table 2:** Sequences of primers used for this study.

Gene	Forward Primer	Reverse Primer
Actin	TTGAATCCCAAGGCGAATAG	TGCGACCACTGGCATAAAG
GR	TGCGAAGTGTTACAAGGCGA	AGAAACTTTGACACATCGAGACG
MDHAR	ACAGCCTTCTTCTGTTGCCTTCAG	CTCTATTGTCGTTGGCGAAATCCG
DHAR	ATGTCGGGCTCCAGA ATCCAACCA	AAAGCGAGGAATTGGAAGGAAGGT
APX	TCACACATTGGGTAGGGCAC	TGCCTTGTCTGATGCCAACT
SOD	GCTGATGGAGTAGCAGAGGC	CCAATCTTCCACCCGCATTG
CAT	ACTTTA AGGAGCCCGGAGAGAG	CGGATAAATCGTTCCTGCCTGTC
POD	TTGTGATGGGTCGGTGCTAC	TGTCCTGATGCCAAGGTGAC
PAO	GGGCATTGAAAACTGGAAGA	TTACTTGGCGATCAAAAATGG
RCCR	TTCGAGTATGGGTAGACGAA	ATCTTGGCAAACTAGAACCC
NYC1	TGATGATATGTTGCCGAGAG	AGTTCTGCCTGTAACGACTT

## Data Availability

Data are contained within the article.
